# Dysregulated miRNA Expression and Its Association with Immune Checkpoints in Head and Neck Cancer

**DOI:** 10.3390/cancers17132169

**Published:** 2025-06-27

**Authors:** Mohd Shuaib, Diksha Saini, Gargi Sharma, Ishwar Singh, Sanjay Gupta, Shashank Kumar, Pramod Kumar

**Affiliations:** 1 Molecular Signaling & Drug Discovery Laboratory, Department of Biochemistry, Central University of Punjab, Guddha, Bathinda 151401, India; shashankbiochemau@gmail.com; 2 Department of Molecular Biology, ICMR-National Institute of Cancer Prevention and Research, Noida 201301, India; diksha98saini@gmail.com; 3 Department of Microbiology, Chaudhary Charan Singh University, Meerut 250001, India; gargi.sharma.bsc.2018@miet.ac.in; 4 Department of ENT, Lok Nayak Hospital, New Delhi 110002, India; drisingh62@gmail.com; 5 Department of ENT, Maulana Azad Medical College, New Delhi 110002, India; 6 Department of Urology, School of Medicine, Case Western Reserve University, Cleveland, OH 44106, USA; sanjay.gupta@case.edu

**Keywords:** head and neck cancer, immune checkpoints, immune evasion, MicroRNAs, immune signaling pathways

## Abstract

**Background**: Head and neck cancer (HNC) remains a global health challenge with a poor 5-year survival rate among patients with relapsed or advanced-stage disease. Immune checkpoint blockade therapies have emerged as a promising approach to improve outcomes; however, their effectiveness is limited, with response rates of only 15–20% because of immune evasion mechanisms. MicroRNA (miRNA) dysregulation plays a key role in facilitating such immune evasion. In this study, we aim to identify specific miRNAs whose altered expression contributes to immune escape in HNC. **Methods**: We employed an integrated bioinformatics approach, incorporating differential expression analysis, survival analysis, target prediction, KEGG immune pathway analysis, a protein–protein interaction network, and the identification of hub genes using in silico tools. **Results**: Our analysis revealed that a high expression of miR-18a and miR-2355 was associated with reduced survival, with the median survival decreasing from 42.9 to 27.8 months, respectively, in advanced-stage patients. Conversely, a low expression of let-7c and miR-6510 was linked to poor prognosis, with survival decreasing from 40.1 to 19.2 months and from 50.1 to 26.8 months, respectively, across disease progression. Further pathway analysis revealed that these miRNAs are significantly involved in the regulation of key immune evasion signaling pathways, including T cell receptor, PD-L1/PD-1 checkpoint, JAK-STAT, TGF-beta, NF-kappa B, and TNF signaling pathways. Hub gene analysis identified AKT1, STAT3, NFKB1, CD4, IL2RB, TLR4, and CTLA-4 as potential dysregulated miRNA targets, with enrichment in immune-related signaling pathways. **Conclusions**: Taken together, these findings suggest that targeting these miRNAs could modulate immune evasion mechanisms and potentially enhance the efficacy of ICB therapies in HNC.

## Simple Summary

The management of HNC is a major global challenge, with chemo-radiotherapy resistance and a low 5-year survival rate. Recently, the FDA has approved anti-PD1/PDL1-based immunotherapies for the better management and longer survival of HNC patients. However, immune-escaping mechanisms mitigate the antitumoral effect of anti-PD-1/PD-L1-based therapies. Dysregulated miRNAs are increasingly recognized as key players in immune evasion mechanisms through the modulation of several immune signaling pathways that are essential for antitumor immune responses. In this study, we aimed to identify miRNAs whose dysregulation is closely associated with immune-escaping mechanisms and lower survival rates in HNC patients. The present study found some key miRNAs that are associated with lower survival rates and immune evasion mechanisms. Targeting the identified miRNAs can help modulate immune evasion mechanisms and possibly enhance the efficacy of anti-PD-1/PD-L1 therapies, including other immune checkpoint therapies in HNC.

## 1. Introduction

Head and neck cancer (HNC) remains one of the most challenging cancers globally, with over 800,000 new cases and approximately 50% mortality annually [1]. Standard treatment options include surgery, radiotherapy, and chemotherapy, administered either alone or in combination [2]. However, despite surgical intervention, treatment outcomes remain limited due to factors such as resistance to chemoradiation, tumor aggressiveness, high heterogeneity, and immune evasion, with the latter being a major contributor to therapeutic failure in HNC [3]. Immunotherapy has significantly advanced cancer treatment, including for HNC. In particular, FDA-approved immune checkpoint inhibitors targeting PD-1/PDL-1 and CTLA-4 have demonstrated substantial progress in treatment strategies [4]. Nevertheless, only a subset of HNC patients, approximately 15–20%, exhibit a complete response, while the majority show partial or no response, limiting the overall survival rate for immune checkpoint blockade (ICB) therapy [5]. This underscores an urgent need to improve the efficacy of ICB therapies in HNC.

The involvement of dysregulated microRNAs (miRNAs) in tumorigenesis, metastasis, and immune modulation is well documented [6,7]. miRNAs are a class of small non-coding RNAs that regulate key cellular processes such as proliferation, differentiation, and apoptosis. The abnormal expression of these miRNAs has been implicated in the initiation and progression of various diseases, including cancer. In HNC, several miRNAs have been associated with disease development and progression through their regulation of tumor suppressors and immune-related genes. Specifically, the overexpression of miR-21, miR-24, miR-31, miR-184, miR-211, and miR-222 and the downregulation of miR-203, miR-100, miR-133, miR-375, miR-145, miR-218, miR-186, miR-191, miR-333, and miR-34a have been linked to HNC pathogenesis [8,9,10]. Growing evidence also highlights the role of dysregulated miRNAs in immune evasion, particularly through the modulation of PD-1/PDL-1 expression and the impairment of the antitumor activity of immune cells such as T cells, macrophages, and dendritic cells [11,12]. For example, preclinical studies have shown that dysregulated expression of miR-21 and miR-155 suppresses the antitumor immune response by modulating the T functions of these immune cells in HNC [13,14]. Conversely, the restoration of miR-34a and miR-146a expression has been demonstrated to regulate PD-L1 levels and the PD-1 checkpoint pathway by modulating PD-L1 expression in tumor cells in HNC [12,15].

However, the existing literature indicates that miRNA dysregulation in HNC contributes to immune evasion and plays a critical role in resistance to immunotherapy, particularly ICB therapy. Therefore, it is essential to identify specific miRNAs whose altered expression drives immune evasion and impairs the effectiveness of ICB therapy in HNC. In this study, we investigated the dysregulated expression of miRNAs implicated in the regulation of key immune evasion signaling pathways in HNC.

## 2. Materials and Methods

### 2.1. Identification and Survival Analysis of Differentially Expressed miRNAs in HNC

Differentially expressed miRNAs in primary HNC tumors were identified using the TCGA dataset via the UALCAN online tool [16]. Their expression patterns were further analyzed across HNC stages (stage 1: *n* = 26; stage 2: *n* = 71; stage 3: *n* = 75; stage 4: *n* = 242) compared to adjacent normal tissues (*n* = 44).

To assess their prognostic relevance, overall survival analysis was performed using the Kaplan–Meier Plotter Database (KMPD) on TCGA samples (primary tumors: *n* = 523; advanced stages: *n* = 317). KMPD utilizes Kaplan–Meier and log-rank tests through the miRpower platform, integrating data from METABRIC, TCGA, and GEO series [17].

### 2.2. Immune Regulation Analysis of miRNAs

Target genes of candidate miRNAs were predicted using TargetScan, which applies a seed-based matching algorithm to identify miRNA–mRNA interactions, retaining targets with at least a “7mer-m8” site [18]. These targets were then subjected to functional enrichment analysis using the DAVID database [19], focusing on immune-related KEGG signaling pathways involved in immune evasion. DAVID evaluates gene lists for enrichment in Gene Ontology (GO) categories—biological processes (BPs), cellular components (CCs), and molecular functions (MFs)—as well as KEGG pathways. Statistical significance was determined using the Benjamini–Hochberg correction to control the false discovery rate (FDR), with pathways showing *p* < 0.05 considered significant.

### 2.3. Protein–Protein Interaction Analysis and Hub Gene Identification

The miRNA target genes involved in statistically significant (*p* < 0.05) immune evasion pathways were used to construct a protein–protein interaction (PPI) network through the STRING database [https://string-db.org/]. The network was built with an interaction score of >0.4 (medium confidence) and active interaction sources. Cytoscape software (v3.38.1) and the cytoHubba app were used for PPI network analysis, with the “Maximal Clique Centrality” (MCC) method applied to identify key hub genes. Hub genes were ranked based on node degree (≥30), and centralities (Closeness and Betweenness) were calculated for further validation.

### 2.4. Expression Validation of miRNAs from GSE Datasets

miRNAs associated with survival and progression were used to validate their expression regulation in gene expression omnibus (GSE) datasets. For this, we searched the HNC GSE datasets and performed a differential expression analysis of the GSE datasets GSE98463 and GSE33299.

### 2.5. Regulatory miRNA–mRNA Correlation and Immune Cell Infiltration Analysis in HNC

A list of selected miRNA target genes from immune evasion signaling pathways were subjected to miRNA–mRNA regulatory analysis. For this, co-expression analysis, with the statistical criteria of a Pearson correlation coefficient (r) < −0.2 and *p*-value < 0.05, was performed for each gene with its regulatory miRNA from the TCGA-HNC dataset using the ENCORI Pan-Cancer Analysis Platform in order to study the negative regulation of target genes. Further, a subset of negatively regulated target genes were used to investigate immune cell infiltration patterns in the tumor immune microenvironment in HNC (*n* = 526) using several dynamics algorithms like CIBERSORT, QUANTISEQ, and XCELL via the TIMER V.3.0.platform.

### 2.6. Statistical Analysis

The Benjamini–Hochberg algorithm was used to identify the false discovery rate in miRNA expression data. GraphPad prism 5.0 software was used for statistical analysis. *p* < 0.05 was used as the statistical significance cutoff across the data.

## 3. Results

### 3.1. Upregulated miRNA Expression and Its Inverse Correlation with Survival in HNC

**Figure 1:**
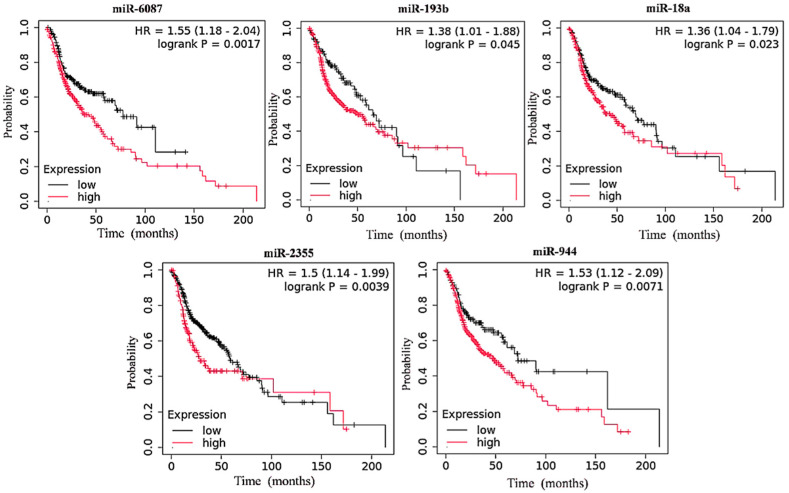
Kaplan–Meier survival analyses of upregulated miRNAs in HNC patient samples over 200 months. A statistically significant log-rank *p*-value > 0.05 and Hazard Ratio (HR) < 1.0 were considered statistically significant factors.

**Figure 2:**
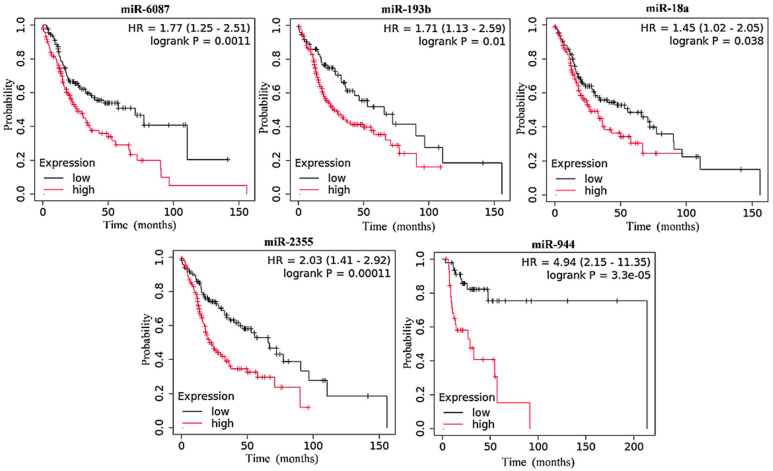
Kaplan–Meier survival analyses of upregulated miRNAs in HNC patient samples over 200 months. The overall survival analysis was performed for advanced stages of HNC. A statistically significant log-rank *p*-value > 0.05 and Hazard Ratio (HR) < 1.0 were considered statistically significant factors.

A total of 40 miRNAs (the top 20 upregulated and the top 20 downregulated miRNAs) were selected from the HNC dataset in the TCGA database (Supplementary Figure S1). The top 20 upregulated miRNAs were analyzed for their association with overall survival across all stages of HNC, including advanced stages, and for their differential expression in the primary stages and various other stages of the disease. The results showed that a high expression of hsa-miR-6087, hsa-miR-193b, hsa-miR-18a, hsa-miR-2355, and hsa-miR-944 was significantly associated with poor survival in all stages of HNC (Figure 1). Notably, the overexpression of these miRNAs was even more strongly associated with reduced survival in advanced stages of HNC (Figure 2).

**Figure 3:**
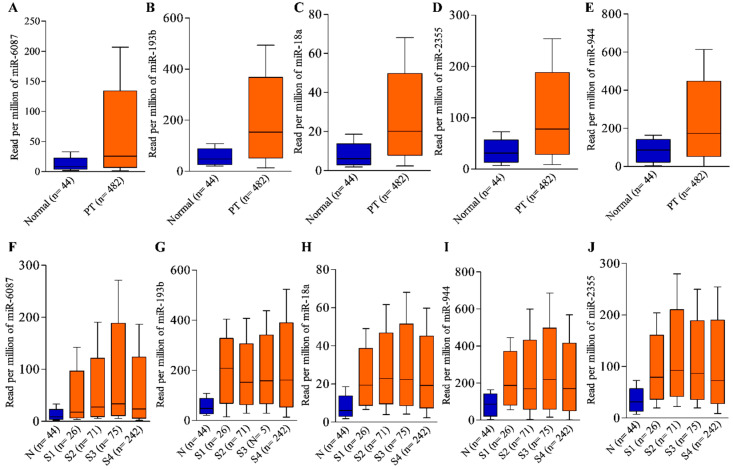
Differential expression of upregulated miRNAs in HNC samples from TCGA database. The expression of (**A**) hsa-miR-6087, (**B**) hsa-miR-193b, (**C**) hsa-miR-18a, (**D**) hsa-miR-2355, and (**E**) hsa-miR-944 in the primary tumor of HNC is shown. Similarly, the differential expression of (**F**) hsa-miR-6087, (**G**) hsa-miR-193b, (**H**) hsa-miR-18a, (**I**) hsa-miR-944, and (**J**) hsa-miR-2355 across the different stages of HNC is shown. The statistically significant *p*-values for each comparison involving hsa-miR-6087 are given [*p* = 1.94 × 10^−12^ (N vs. PT), *p* = 1.52 × 10^−1^ (N vs. S1), *p* = 8.95 × 10^−4^ (N vs. S2), *p* = 1.37 × 10^−3^ (N vs. S3), *p* = 8.36 × 10^−8^ (N vs. S4)], hsa-miR-193b [*p* = 1.62 × 10^−12^ (N vs. PT), *p* = 2.78 × 10^−7^ (N vs. S1), *p* = 5.09 × 10^−12^ (N vs. S2), *p* = 4.68 × 10^−14^ (N vs. S3), *p* = 1.62 × 10^−12^ (N vs. S4)], hsa-miR-18a [*p* = 1.62 × 10^−12^ (N vs. PT), *p* = 2.21 × 10^03^ (N vs. S1), *p* = 4.99 × 10^−10^, (N vs. S2) *p* = 8.34 × 10^−11^ (N vs. S3), *p* < 1 × 10^−12^ (N vs. S4)], hsa-miR-2355 [*p* < 1 × 10^−12^ (N vs. PT), *p* = 5.02 × 10^−4^ (N vs. S1), *p* = 5.54 × 10^−14^ (N vs. S2), *p* = 9.67 × 10^−12^ (N vs. S3), *p* = 1.11 × 10^−16^ (N vs. S4)], hsa-miR-944 [*p* = 1.62 × 10^−12^ (N vs. PT), *p* = 1.75 × 10^−5^ (N vs. S1), *p* = 2.49 × 10^−10^ (N vs. S2), *p* = 9.92 × 10^−10^ (N vs. S3), *p* = 1 × 10^−12^ (N vs. S4)]. N: normal; PT: primary tumor; S1: stage 1; S2: stage 2; S3: stage 3; and S4: stage 4.

The differential expression of these miRNAs was notably higher in advanced stages of HNC compared to primary tumors (Figure 3). In the low-expression cohort, the median survival time ranged from 58.7 to 77.3 months across all stages (Table 1), while the high-expression cohort had a median survival time ranging from 27.8 to 50.1 months (Table 1). In advanced stages, the high-expression cohort showed a survival time range of 22.67 to 32.67 months, while the low-expression cohort had a median survival time ranging from 55.70 to 70.67 months (Table 1). In addition, data for the best cutoff values and expression ranges for each miRNA in combined stages across HNC is provided in Supplementary Table S3.

### 3.2. Top Downregulated miRNAs and Their Prognostic Potential in HNC

**Figure 4:**
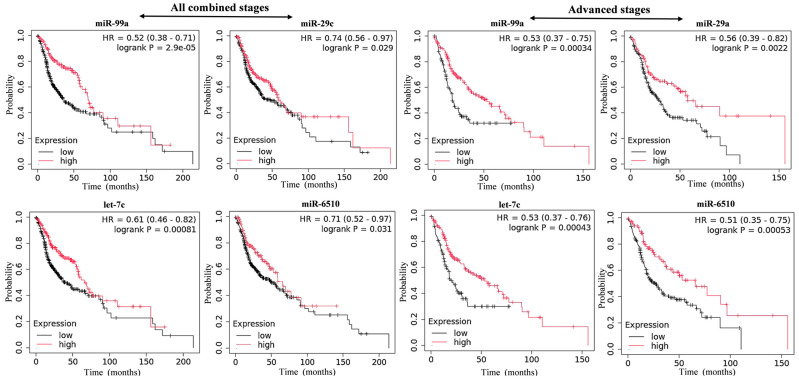
Kaplan–Meier survival analyses of downregulated miRNAs in all samples of combined and advanced stages of HNC from TCGA database over 200 months. A statistically significant log-rank *p*-value > 0.05 and Hazard Ratio (HR) < 1.0 were considered statistically significant factors.

**Figure 5:**
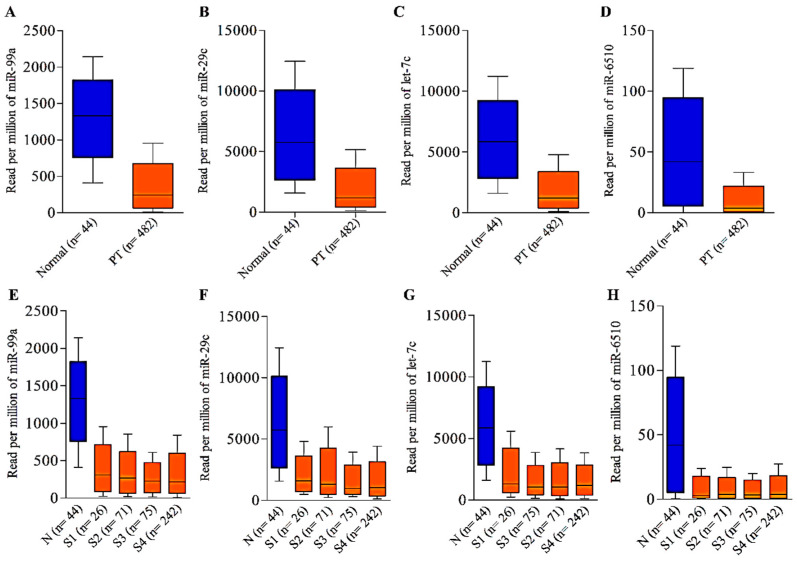
Differential expression of downregulated miRNAs in HNC samples from TCGA database. The expression of (**A**) hsa-miR-99a, (**B**) hsa-miR-29c, (**C**) hsa-let-7c, and (**D**) hsa-miR-6510 in the primary tumor of HNC is shown. Similarly, the differential expression of (**E**) hsa-miR-99a, (**F**) hsa-miR-29c, (**G**) hsa-let-7c, and (**H**) hsa-miR-6510 across the different stages of HNC is shown. The statistically significant *p*-values are hsa-miR-6510: *p* = 5.96 × 10^−5^ (N vs. PT), *p* = 4.86 × 10^−6^ (N vs. S1), *p* = 2.18 × 10^−4^ (N vs. S2), *p* = 1.81 × 10^−4^ (N vs. S3), and *p* = 6.00 × 10^−7^ (N vs. S4); hsa-let-7c *p* = 1.11 × 10^−16^ (N vs. PT), *p* = 3.65 × 10^−12^ (N vs. S1), *p* = 1.65 × 10^−12^ (N vs. S2), *p* = 2.70 × 10^−12^ (N vs. S3), and *p* = 1.13 × 10^−14^ (N vs. S4); hsa-mir-29c: *p* = 1.59 × 10^−9^ (N vs. PT), *p* = 4.65 × 10^−9^ (N vs. S1), *p* = 1.47 × 10^−7^ (N vs. S2), *p* = 2.66 × 10^−10^ (N vs. S3), and *p* = 4.19 × 10^−10^ (N vs. S4); and hsa-mir-99a: *p* < 1 × 10^−12^ (N vs. PT), *p* = 2.86 × 10^−12^ (N vs. S1), *p* = 6.66 × 10^−16^ (N vs. S2), *p* = 2.57 × 10^−11^ (N vs. S3), and *p* = 1.62 × 10^−12^. N: normal; PT: primary tumor; S1: stage 1; S2: stage 2; S3: stage 3; and S4: stage 4.

The top 20 downregulated miRNAs were analyzed for their prognostic value in HNC progression. These miRNAs were examined for their association with overall survival and their expression pattern in primary- and advanced-stage HNC samples. The results showed that the downregulation of hsa-miR-99a, hsa-miR-29c, hsa-let-7c, and hsa-miR-6510 was significantly associated with poor survival across all stages, particularly advanced stages of HNC (Figure 4). Additionally, the downregulation of these miRNAs was observed across different stages of HNC (Figure 5). In the low-expression cohort, the median survival times ranged from 36.03 to 50.13 months (Table 1), while in the high-expression cohort, the median survival times were inversely correlated, ranging from 58.7 to 69.4 months (Table 1). In the advanced stages, the low-expression cohort had median survival times between 18.20 and 27.97 months, whereas the high-expression cohort showed a median survival range of 53.03 to 65.73 months. (Table 1). The data for the best cutoff values and expression ranges for each miRNA in advanced stages of HNC is provided in Supplementary Table S3.

### 3.3. Enrichment of Candidate miRNAs in Immune Evasion

To investigate the role of key upregulated miRNAs (hsa-miR-6087, hsa-miR-193b, hsa-miR-18a, hsa-miR-2355, and hsa-miR-944) and downregulated miRNAs (hsa-miR-99a, hsa-miR-29c, hsa-let-7c, and hsa-miR-6510) in immune evasion in HNC, we performed functional enrichment analysis. Target genes for each miRNA were predicted using TargetScan, identifying 13,817 target genes for upregulated miRNAs (Supplementary Table S1) and 8676 for downregulated miRNAs (Supplementary Table S2). The predicted target genes of hsa-miR-18a and hsa-miR-2355 (upregulated) and hsa-let-7c and hsa-miR-6510 (downregulated) were significantly enriched (*p* < 0.05) in key immune evasion pathways, including T cell receptor signaling, PD-L1 expression, and PD-1 checkpoint, JAK-STAT, TGF-beta, B cell receptor, NF-kappa B, and TNF signaling pathways (Table 2).

### 3.4. Protein–Protein Interaction (PPI) Analysis and Hub Gene Identification in Immune Evasion

**Figure 6:**
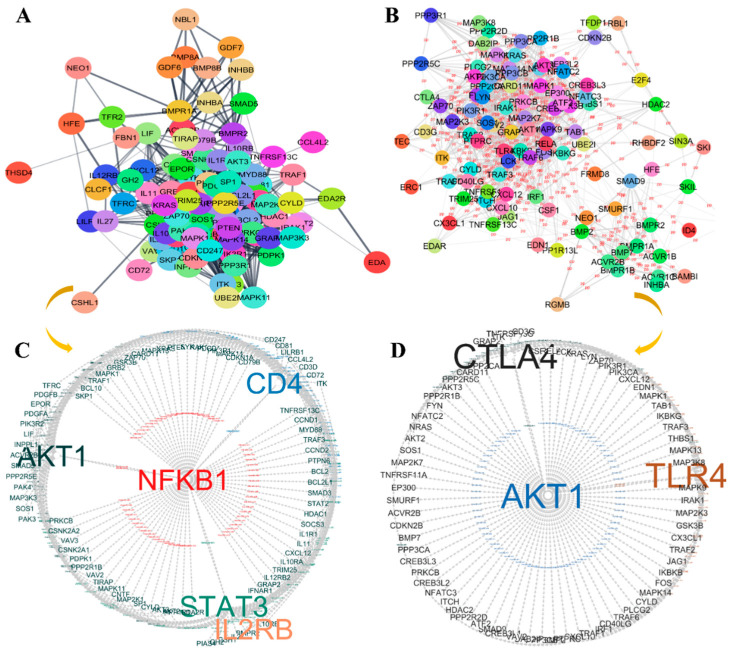
Protein–protein interaction (PPI) and hub gene analysis across immune-related genes in HNC. (**A**) PPI network of upregulated and (**B**) downregulated miRNA target genes involved in key immune evasion signaling pathways. (**C**) Visualization of protein–protein associations of hub genes belong to upregulated miRNAs. (**D**) Visualization of protein–protein associations of hub genes belong to downregulated miRNAs.

The upregulated and downregulated miRNAs’ predicted genes were enriched in key immune evasion pathways and analyzed for their PPI information using the STRING database. The PPI network of upregulated miRNAs contained 105 nodes, with an average node degree of 18.70, enriched in 980 edges at a statistically significant *p*-value < 1.0 × 10^−16^ (Figure 6A). Similarly, the downregulated miRNAs’ PPI network had 101 nodes, with an average node degree of 18.20, significantly enriched in 921 edges (*p* < 1.0 × 10^−16^) (Figure 6B). Cytoscape software was used to analyze both networks and identify hub genes. Genes with the highest degree score (≥30), along with closeness and betweenness centralities, were selected as potential hub genes using the cytoHubba plugin’s MCC method (Table 3). The protein–protein associations of these hub genes were visualized in Cytoscape (Figure 6C,D). AKT1, STAT3, NFKB1, CD4, and IL2RB were identified as key immune signature hub genes for upregulated miRNAs (Figure 6C and Table 3), while AKT1, TLR4, and CTLA-4 were identified as signature hub genes for downregulated miRNAs (Figure 6D and Table 3).

### 3.5. Expression Validation of Leading miRNAs Using GSE Dataset

The present study identified miR-18a, miR-2355, miR-let-7c, and miR-6510 as leading miRNAs. The expression of miR-18a and miR-2355 was upregulated (Figure 3C,D), while miR-let-7c and miR-6510 were downregulated in HNC (Figure 5C,D). More interestingly, in the expression validation analysis of these miRNAs, this study found similar expression regulation of miR-18a, miR-2355, miR-let-7c, and miR-6510 in the GSE datasets GSE98463 and GSE33299 for HNC. The expression of miR-18a (Log2FC = +0.48) and miR-2355 (Log2FC = +0.15) was upregulated in GSE33299 and GSE98463, respectively. Similarly, the expression of miR-let-7c (Log2FC = −0.35) and miR-6510 (Log2FC = −3.57) was downregulated in the GSE33299 and GSE98463 datasets for HNC, respectively.

### 3.6. The miRNA–mRNA Co-Expression Analysis and Immune Cell Infiltration Patterns in HNC

**Figure 7:**
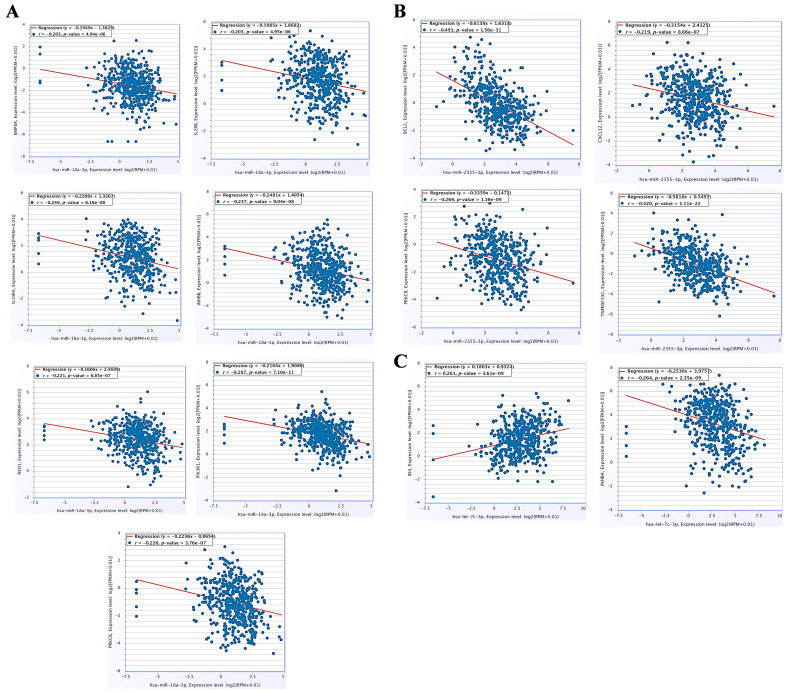
The miRNA–mRNA co-expression analysis in HNC. (**A**) Co-expression between miR-181a-5p and its target genes BMP8A, IL2RB, IL10RB, INHBB, NEO1, PIK3R1, and PRKCB. (**B**) Co-expression between miR-2355-3p and its target genes BCL2, CXCL12, PRKCB, and TNFRSF13C. (**C**) Co-expression between let-7c-3p and its target genes ID4 and INHBA.

The co-expression analysis of the target genes of miR-18a-3p, miR-2355, let-7c-3p, and miR-6510-5p significantly involved in immune evasion signaling pathways was utilized to understand their regulation in HNC. As a result, statistically significant negative regulation was found in HNC between miR-18a-3p and BMP8A, IL2RB, IL10RB, INHBB, NEO1, PIK3R1, and PRKCB. (Figure 7A); miR-2355-3p and BCL2, CXCL12, PRKCB, and TNFRSF13C (Figure 7B); and let-7c-3p and ID4 and INHBA (Figure 7C). However, the present study did not find any statistically significant negative correlations between miR-6510-5p and its target genes. Furthermore, these leading target genes were subjected to immune cell infiltration analysis in order to determine their potential involvement in shaping tumor immune microenvironments in HNC. The results showed the strong association of target gene expression with the significant infiltration of tumor-targeting immune cells such as macrophages, CD8^+^ T cells, CD4^+^ T cells, and myeloid dendritic cells. A Spearman correlation coefficient (ρ) greater than 0.4 with a statistically significant *p*-value less than 0.05 represents a strong correlation between gene expression and immune cell infiltration in HNC (Supplementary Table S4).

## 4. Discussion

The management of HNC continues to be a major challenge due to its aggressive nature, high rates of metastasis, resistance to treatment, and recurrence. Immune evasion plays a pivotal role in treatment resistance and disease recurrence. Dysregulated miRNAs are increasingly recognized as key players in immune evasion by modulating pathways such as PD-1/PD-L1 expression and the activity of immune cells like T cells, macrophages, and dendritic cells [11,12], which are essential for antitumor immune responses. When these cells are manipulated by tumor cells, immune evasion ensues, contributing significantly to tumor progression.

In this study, we identified miR-18a, miR-2355, let-7c, and miR-6510 as important regulators of immune evasion signaling pathways in HNC progression. The differential expression analysis of the TCGA HNC dataset revealed that miR-18a and miR-2355 were upregulated while let-7c and miR-6510 were downregulated in primary tumor samples compared to adjacent normal tissues. These dysregulated miRNAs exhibited increased expression across all stages of HNC, suggesting a close association with disease progression. The survival analysis further indicated that the overexpression of miR-18a and miR-2355 correlates with poor survival across all stages, with the worst survival outcomes in advanced stages. These findings align with previous studies, which also reported that elevated levels of miR-18a are linked to poor survival and enhanced tumor progression in HNC [3,20,21,22,23]. Similarly, miR-2355 has been shown to promote cell proliferation, invasion, and metastasis in other cancers, underscoring its potential as an oncogenic miRNA in HNC [24,25]. In contrast, let-7c and miR-6510 were downregulated in primary tumors and across all stages of HNC. The reduced expression of these miRNAs was inversely correlated with survival, with further decreases in their expression during advanced stages of HNC, resulting in significantly worse survival outcomes. This supports findings from previous studies, which have shown that let-7c acts as a tumor suppressor and its downregulation contributes to HNC progression [25,26,27]. Moreover, restoring let-7c expression has been proposed as a potential therapeutic strategy to enhance the efficacy of chemotherapy [26,27]. The downregulation of miR-6510, whose functional role in HNC is largely unexplored, presents an opportunity for further investigation to uncover its role in HNC progression and treatment resistance.

While the role of miR-18a, miR-2355, let-7c, and miR-6510 in immune modulation and HNC progression has been suggested, their precise immunomodulatory roles in HNC and their involvement in immune evasion mechanisms have not been fully explored. To address this, we conducted a functional enrichment analysis and found that the target genes of these dysregulated miRNAs were significantly enriched in several key immune evasion signaling pathways, including the JAK-STAT, T cell receptor signaling, PD-L1 expression, PD-1 checkpoint, TGF-beta signaling, NF-kappa B, and TNF signaling pathways. These pathways are well-established in HNC progression and immune evasion, with many studies highlighting their role in inhibiting immune responses, promoting tumor growth, and contributing to immune checkpoint blockade (ICB) therapy resistance.

For example, the hyperactivation of the JAK-STAT and TGF-beta signaling pathways has been implicated in immune suppression [28,29], while the PD-1/PD-L1 pathway is known to impair T cell activity and promote immune evasion in HNC [30]. Similarly, the dysregulation of NF-kappa B and TNF signaling contributes to the inflammatory tumor microenvironment, further inhibiting antitumor immune responses [31,32]. These findings support the hypothesis that modulating these signaling pathways via their respective miRNAs could potentially overcome immune evasion and enhance the efficacy of ICB therapies in HNC.

In addition, we identified key hub genes such as AKT1, STAT3, NFKB1, CD4, IL2RB, TLR4, and CTLA-4, which were central to the immune-related pathways enriched by the dysregulated miRNAs. These hub genes play critical roles in immune evasion and are involved in resistance to ICB therapies in HNC [33,34,35,36,37]. For instance, the overexpression of AKT1 has been shown to upregulate PD-L1 expression, contributing to resistance to anti-PD-L1 therapy [38,39]. The oncogenic activation of STAT3, NFKB1, and TLR4, along with altered expression of CD4 and IL2RB, further supports immune evasion by enhancing proinflammatory cytokine production and inhibiting cytotoxic T cell activity [36,40,41,42,43]. These findings suggest that targeting these hub genes through their respective miRNAs may serve as a novel therapeutic strategy to mitigate immune evasion and improve the efficacy of ICB therapies in HNC. IC-mediated T cell exhaustion is involved in inhibiting TCR and PI3K–AKT signaling.

A strong correlation was observed between the selected miRNAs (miR-18a-3p, miR-2355, let-7c-3p, and miR-6510-5p) and their target genes (BMP8A, IL2RB, IL10RB, INHBB, NEO1, PIK3R1, PRKCB BCL2, CXCL12, PRKCB, TNFRSF13C, ID4, and INHBA), which were significantly enriched in immune evasion signaling pathways. Therefore, such an miRNA–mRNA correlation analysis revealed that the expression regulation of these genes via their respective miRNAs can modulate immune evasion in HNC. Furthermore, the present study found a strong correlation between the expression of target genes (IL10RB, INHBB, NEO1, PIK3R1, BCL2, ID4, and INHBA) and the infiltration of tumor-killing cells into the tumor immune microenvironment in HNC. The immune infiltration analyses revealed the infiltration of immune cells, particularly CD8^+^ T cells, CD4^+^ T cells, dendritic cells, and natural killer cells, in HNC. CD8+ is activated in CTLs and mediates the effector function through killing tumor cells in the TME. However, the mechanisms involved in maintaining an immune-suppressive TME and one of the important underlying mechanisms in the overexpression of the negative regulation of ICs, including PD1/CTLA-4, dampen the immune response. Despite immune infiltration, other mechanisms such as DC conditioning, M1-to-M2 macrophage polarization, and the overexpression of ICs in immune effector cells dampen the antitumoral efficacy, which leads to the progression of HNC. Moreover, several studies have reported that the overexpression of these genes is significantly involved in immune escape mechanisms by generating an immunosuppressive tumor microenvironment and proinflammatory cytokines, reducing T cell activation, and favoring Treg maintenance in several cancers [44,45,46,47]. Our results are in line with previous findings and indicate that the modulation of these genes’ expression via their respective miRNAs may enhance the immune response and modulate the immune escape mechanism via the infiltration of tumor-killing cells into the HNC tumor microenvironment.

## 5. Conclusions

In conclusion, the dysregulated expression of miR-18a, miR-2355, let-7c, and miR-6510 in HNC plays a significant role in immune evasion and tumor progression. Targeting these miRNAs and their associated immune signaling pathways offers promising avenues for therapeutic intervention. Moreover, the identification of key genes in the tumor immune microenvironment (TIMR), such as IL10RB, INHBB, NEO1, PIK3R1, BCL2, ID4, and INHBA, further underscores the potential of miRNA-based therapies to enhance the efficacy of ICB treatments in HNC, providing a new direction for improving clinical outcomes in patients with this challenging cancer.

## Figures and Tables

**Table 1:** Median survival of HNC patients in cohorts with low and high expression of miRNAs.

miRNA	Low-Expression Cohort (Months)		High-Expression Cohort (Months)	
	All Combined Stages	Advance Stages	All Combined Stages	Advance Stages
**Overexpressed**				
hsa-miR-6087	77.30	70.67	37.77	26.30
hsa-miR-193b	65.73	65.73	50.13	26.87
hsa-miR-18a	69.43	55.70	42.97	26.87
hsa-miR-2355	58.73	65.73	27.87	22.67
hsa-miR-944	72.20	57.73	46.60	32.67
**Underexpressed**				
hsa-miR-99a	36.03	18.20	69.43	53.03
hsa-miR-29c	47.67	27.97	58.73	57.73
hsa-let-7c	40.07	19.23	65.73	53.03
hsa-miR-6510	50.13	26.87	65.73	65.73

**Table 2:** MiRNAs’ functional involvement in key immune signaling pathways in HNC.

Regulatory miRNA	Immune Signaling Pathways	Gene Count	*p*-Value	Genes Involved
**Upregulated miRNAs’ signaling pathways**				
miR-18a-3p	hsa04630:JAK-STAT signaling pathway	33	0.023954886	CDKN1A, CNTF, CSH1, PDGFB, PDGFA, PIK3R2, IL27, PIK3R1, SOCS3, CCND2, CCND1, AKT2, AKT3, AKT1, IL12RB2, PIAS4, IL11, IL10RB, IL10RA, STAT2, STAT3, LIF, CSHL1, EPOR, GH2, CLCF1, IL2RB, BCL2, GRB2, PTPN6, SOS1, BCL2L1, IFNAR1
	hsa04660:T cell receptor signaling pathway	30	0.001266955	GSK3B, ITK, PIK3R2, PIK3R1, CD3D, PPP3R1, PPP2R1B, PPP2R5E, AKT2, GRAP2, AKT3, AKT1, MAPK1, PAK3, PAK4, VAV3, MAP2K1, PDPK1, MAPK14, MAPK13, VAV2, MAPK11, CD4, GRB2, KRAS, PTPN6, PDCD1, CD247, SOS1, CARD11
	hsa04350:TGF-beta signaling pathway	23	0.0279178	BMPR2, SMAD3, TFRC, HDAC1, HFE, BMP8A, BMP8B, INHBB, INHBA, GDF6, SMAD5, ACVR2B, THSD4, GDF7, PPP2R1B, SP1, TFR2, MAPK1, NBL1, NEO1, SKP1, BMPR1A, FBN1
	hsa05235:PD-L1 expression and PD-1 checkpoint pathway in cancer	22	0.008014647	MAP3K3, MAP2K1, CSNK2A2, STAT3, PTEN, PIK3R2, PIK3R1, MAPK14, CD3D, MAPK13, MAPK11, CD4, PPP3R1, AKT2, AKT3, AKT1, MAPK1, KRAS, PTPN6, PDCD1, CD247, MYD88
	hsa04662:B cell receptor signaling pathway	22	0.007028352	VAV3, GSK3B, MAP2K1, CD72, PRKCB, CD81, INPPL1, LILRB1, PIK3R2, PIK3R1, VAV2, CD79B, PPP3R1, AKT2, AKT3, AKT1, MAPK1, GRB2, KRAS, PTPN6, SOS1, CARD11
miR-2335-3p	hsa04064:NF-kappa B signaling pathway	25	0.014080501	EDA, CCL4L1, TNFRSF13C, BCL10, EDA2R, IRAK1, PPP1R13L, TRIM25, PIAS4, EDA, RADD, UBE2I, SYK, CSNK2A1, PRKCB, IL1R1, TRAF1, TIRAP, NFKB1, CYLD, ZAP70, CXCL12, TRAF3, BCL2, MYD88, CARD11
**Downregulated miRNAs’ signaling pathways**				
Let-7c	hsa04350:TGF-beta signaling pathway	29	1.37E-04	HDAC2, BMPR2, ZFYVE9, HFE, ACVR1B, THBS1, PPP2CA, ACVR1C, PPP2R1B, SIN3A, EP300, E2F4, NEO1, SKIL, CDKN2B, SMURF1, SMAD9, INHBA, RGMB, BMP7, ACVR2B, SKI, BMP2, RBL1, TFDP1, BAMBI, ID4, BMPR1B, BMPR1A
	hsa04660:T cell receptor signaling pathway	29	0.001149086	GSK3B, ITK, CD3G, PIK3R1, PPP2CA, PPP3CA, MAPK9, PPP3CB, NRAS, PPP3R1, PPP2R1B, GRAP2, AKT3, CTLA4, FYN, MAP3K8, NFATC3, NFATC2, PPP2R5C, FOS, MAPK14, VAV2, PTPRC, TEC, PIK3CA, LCK, PPP2R2D, KRAS, SOS1
miR-6510	hsa04668:TNF signaling pathway	28	0.02621548	ATF2, CSF1, RELA, CX3CL1, IKBKB, CREB3L3, AKT2, CREB3L1, CREB3L2, AKT1, MAPK1, IKBKG, MAP2K7, MAP2K3, EDN1, JAG1, RHBDF2, DAB2IP, TRAF2, TRAF1, MAPK13, CYLD, CXCL10, ITCH, FRMD8, TRAF3, IRF1, TAB1
	hsa04064:NF-kappaB signaling pathway	25	0.031971598	TNFRSF13C, TNFRSF11A, RELA, IKBKB, IRAK1, PPP1R13L, PLCG2, TRIM25, IKBKG, ERC1, LYN, UBE2I, PRKCB, TRAF2, TRAF1, EDAR, CYLD, ZAP70, CXCL12, CD40LG, TRAF3, TRAF6, TAB1, TLR4, CARD11

**Table 3:** Identified hub genes with highest node degree for up- and downregulated miRNAs involved in immune evasion signaling pathways.

Gene Node	Identifier	Node Degree	Betweenness	Closeness
**Upregulated miRNAs hub genes in immune evasion**				
**AKT1**	9606.ENSP00000451828	65	1151.233	83.83333
**STAT3**	9606.ENSP00000264657	60	970.7170	81.50000
**NFKB1**	9606.ENSP00000226574	58	777.0496	79.25000
**CD4**	9606.ENSP00000011653	53	1003.502	77.66667
BCL2	9606.ENSP00000381185	45	473.0872	73.50000
PIK3R1	9606.ENSP00000428056	43	622.3602	73.00000
PTEN	9606.ENSP00000361021	43	229.4419	71.58333
KRAS	9606.ENSP00000256078	39	228.9372	69.83333
MAPK1	9606.ENSP00000215832	39	176.7926	69.75000
MYD88	9606.ENSP00000498321	38	224.4583	68.91667
SYK	9606.ENSP00000364898	38	151.3794	69.08333
GRB2	9606.ENSP00000376345	37	130.7906	68.58333
GSK3B	9606.ENSP00000324806	35	143.0187	67.58333
MAPK14	9606.ENSP00000229795	34	117.3630	66.91667
PTPN6	9606.ENSP00000391592	32	155.2973	66.08333
BCL2L1	9606.ENSP00000365230	31	140.8044	65.16667
CCND1	9606.ENSP00000227507	31	45.69035	65.41667
**IL2RB**	9606.ENSP00000216223	31	63.51949	65.58333
ZAP70	9606.ENSP00000264972	30	96.13023	64.66667
**Downregulated miRNAs hub genes in immune evasion**				
**AKT1**	9606.ENSP00000451828	69	83.50000	1719.840
RELA	9606.ENSP00000384273	45	71.00000	620.9879
TRAF6	9606.ENSP00000433623	43	70.00000	383.0736
GSK3B	9606.ENSP00000324806	42	69.33333	395.8324
MAPK1	9606.ENSP00000215832	40	68.50000	324.4336
FOS	9606.ENSP00000306245	38	67.00000	235.4975
LCK	9606.ENSP00000477713	37	66.00000	164.1376
**TLR4**	9606.ENSP00000363089	37	66.00000	144.1318
FYN	9606.ENSP00000346671	36	66.33333	248.3050
EP300	9606.ENSP00000263253	35	65.83333	292.4875
KRAS	9606.ENSP00000256078	35	64.66667	155.6484
LYN	9606.ENSP00000428924	35	64.83333	139.4930
PIK3CA	9606.ENSP00000263967	35	66.16667	498.1811
IKBKB	9606.ENSP00000430684	32	63.66667	127.1822
PTPRC	9606.ENSP00000411355	31	63.50000	139.3552
**CTLA4**	9606.ENSP00000497102	30	62.50000	93.57661
IKBKG	9606.ENSP00000483825	30	61.83333	127.4694

Genes in bold are key immune signaling pathway genes.

## Data Availability

The other data that support the findings of this study are available in the Supplementary Materials of this article.

## Supplementary Materials

The following supporting information can be downloaded at https://www.mdpi.com/article/10.3390/cancers17132169/s1, Figure S1: Top 20 up- and downregulated miRNAs in HNC; Table S1: Target genes for upregulated miRNAs; Table S2: Target genes for downregulated miRNAs; Table S3: Cutoff values and expression ranges of miRNAs in patients with combined and advanced stages of HNC; Table S4: Correlation between gene expression and immune cell infiltrations in the tumor microenvironment for HNC.
