# Supplementary material for: Dysregulated miRNA Expression and Its Association with Immune Checkpoints in Head and Neck Cancer
Source: Cancers (Basel). 2025 Jun 27;17(13):2169. doi: 10.3390/cancers17132169 (PMC12249186; doi:10.3390/cancers17132169)
Supplement: Supplementary file 1 [file cancers-17-02169-s001.zip › Table S3. Cutt off values and eexpression ranges of miRNAs in combined and advanced stages of HNC patients..pdf]

Table S3. Cutt off values and eexpression ranges of miRNAs in combined and advanced stages of HNC patients.

miRNA	Cutt off	Expression range	Cutt off	Expression range
	All Combined stages		Advanced stage	
hsa-miR-6807	0	0 - 2	0	0 - 2
hsa-miR-18a	19	2 - 242	19	2 - 242
hsa-miR-193b	93	13 - 1324	100	13 - 1324
hsa-miR-2355	117	8 - 517	69	8 - 517
hsa-miR-944	112	2 - 1462	116	2 - 1239
hsa-miR-99a	375	8 - 5614	174	8 - 3232
hsa-miR-29c	1538	113 - 17765	1496	113 - 9482
hsa-let-7c	1831	96 - 17174	897	106 - 14931
hsa-miR-6510	12	0 - 489	7	0 - 489
