# Supplementary figures and images for: Dysregulated miRNA Expression and Its Association with Immune Checkpoints in Head and Neck Cancer
Source: Cancers (Basel). 2025 Jun 27;17(13):2169. doi: 10.3390/cancers17132169 (PMC12249186; doi:10.3390/cancers17132169)

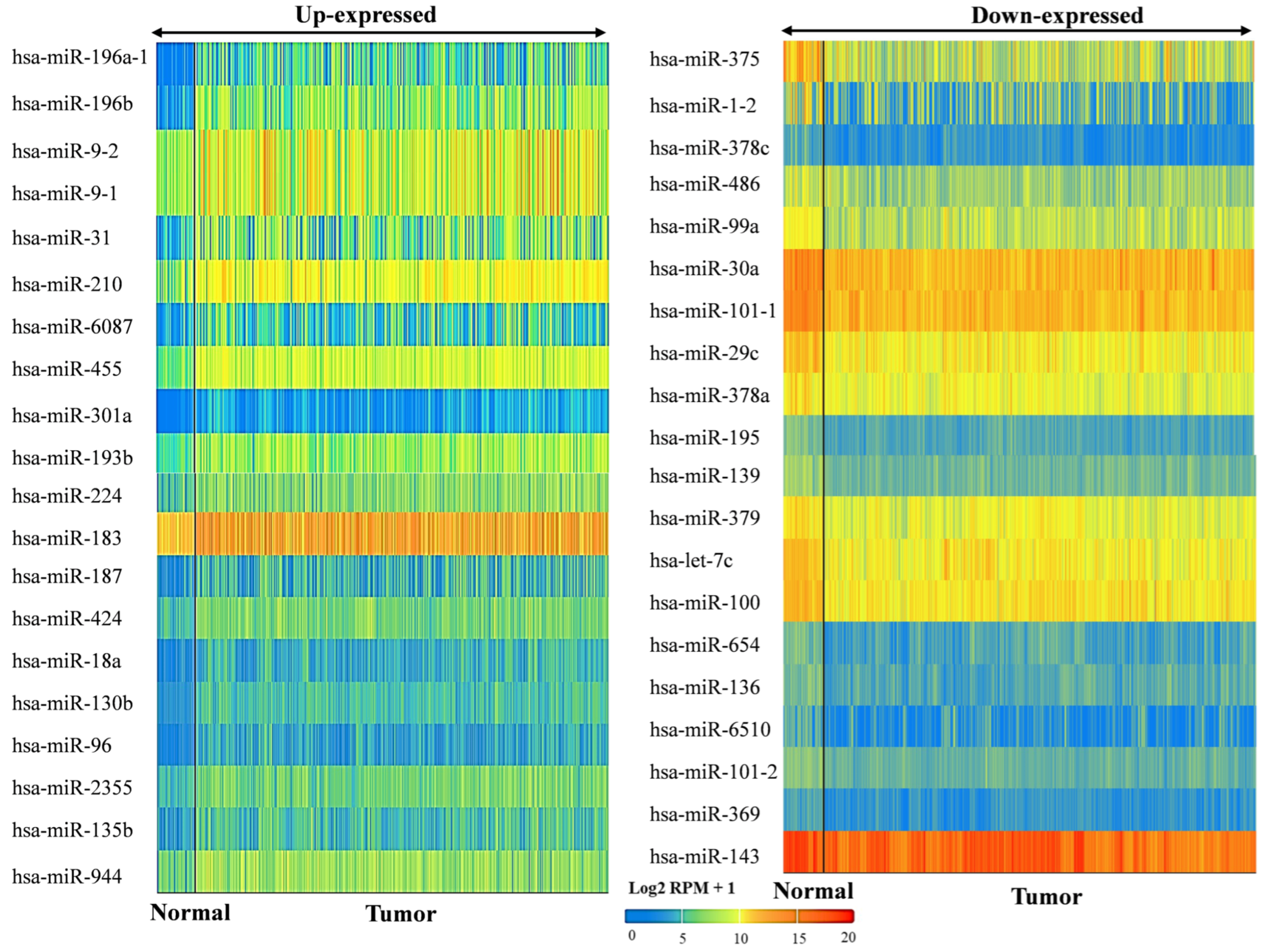

Supplement: Supplementary file 1 [file cancers-17-02169-s001.zip › Figure S1 Top 20 up and down expressed miRNAs in HNC.tif]
